# Using a deep generation network reveals neuroanatomical specificity in hemispheres

**DOI:** 10.1016/j.patter.2024.100930

**Published:** 2024-02-12

**Authors:** Gongshu Wang, Ning Jiang, Yunxiao Ma, Dingjie Suo, Tiantian Liu, Shintaro Funahashi, Tianyi Yan

**Affiliations:** 1 School of Medical Technology, Beijing Institute of Technology, Beijing 100081, China; 2 Advanced Research Institute for Multidisciplinary Science, Beijing Institute of Technology, Beijing 100081, China; 3 Department of Cognitive and Behavioral Sciences, Graduate School of Human and Environmental Science, Kyoto University, Sakyo-ku, Kyoto 606-8501, Japan; 4 Kokoro Research Center, Kyoto University, Sakyo-ku, Kyoto 606-8501, Japan

**Keywords:** hemispherical lateralization, deep generative network, self-supervised learning, MRI

## Abstract

Asymmetry is an important property of brain organization, but its nature is still poorly understood. Capturing the neuroanatomical components specific to each hemisphere facilitates the understanding of the establishment of brain asymmetry. Since deep generative networks (DGNs) have powerful inference and recovery capabilities, we use one hemisphere to predict the opposite hemisphere by training the DGNs, which automatically fit the built-in dependencies between the left and right hemispheres. After training, the reconstructed images approximate the homologous components in the hemisphere. We use the difference between the actual and reconstructed hemispheres to measure hemisphere-specific components due to asymmetric expression of environmental and genetic factors. The results show that our model is biologically plausible and that our proposed metric of hemispheric specialization is reliable, representing a wide range of individual variation. Together, this work provides promising tools for exploring brain asymmetry and new insights into self-supervised DGNs for representing the brain.

## Highlights

• Deep generative network (DGN) is linked to the principles of hemispheric lateralization
• A map between homologous components of the left and right hemispheres is established
• Output errors of DGN represent hemisphere-specific components of the brain
• The proposed method captures a wide range of individual variation

## The bigger picture

The brain exhibits clear structural and functional asymmetries. To better understand the intrinsic properties of the brain, it is important to estimate the specificity of each hemisphere despite the complexity of its organization. Deep generative networks (DGNs) are a type of machine learning model that offers powerful inference and recovery capabilities. DGNs can be leveraged to model brain development and formation and estimate hemisphere-specific components. The application of DGNs for brain representation could advance neuroscience research and specifically help us understand hemispheric specialization.

## Graphical abstract

**Figure:**
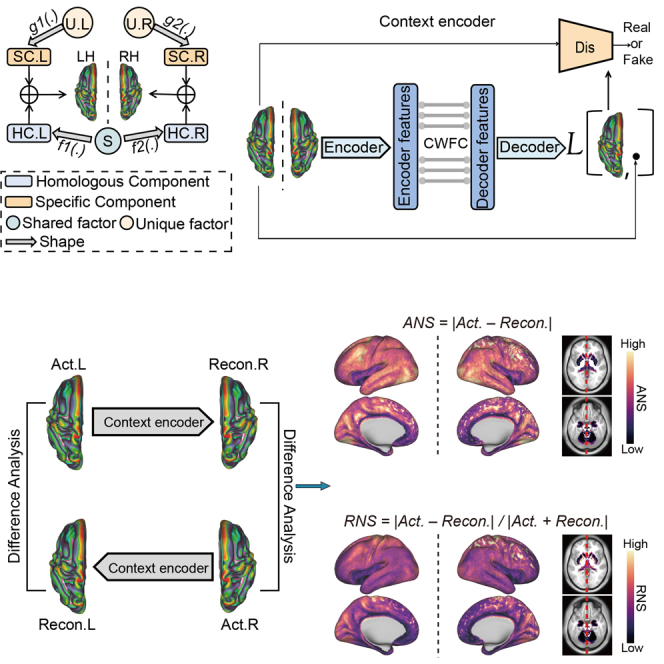

## Teaser

Understanding the components unique to each hemisphere of the brain is crucial for investigating the mechanisms of brain development and asymmetry. This study connects the computational principles of deep generative networks to the formation of the brain’s hemispheres and shows that the differences between reconstructed and actual hemispheres can be utilized to identify hemisphere-specific components.

## Introduction

Although the left hemisphere (LH) and right hemisphere (RH) of our brains develop with a high degree of symmetry at both the anatomical and functional levels, it has become clear that the two sides develop and age at different rates, have subtle structural and functional differences, and are each dominant in processing specific cognitive and perceptual tasks.^1^^,^^2^^,^^3^ A complex and ongoing interplay of genetic and environmental factors most likely underlies and guides this lateralized specialization. Genes provide templates for creating brain patterns that probably trigger only modest hemispheric dominance, while prenatal and postnatal environments shape and influence the orientation of emerging neural networks, reinforcing hemispheric dominance.^4^^,^^5^^,^^6^ Additionally, brain asymmetries also depend on interhemispheric interactions, which are supported by the corpus callosum.^7^ Stronger bilateral interregional communication facilitates interhemispheric sharing, whereas weaker bilateral interregional communication results in hemispheric uniqueness.

Understanding the nature of brain asymmetry and how it contributes to individual variation is an enduring crucial topic in neuroscience. Previous studies have revealed widespread neuroanatomical asymmetries in the brain that occur across cortical and subcortical volumes as well as cortical thickness and surface area.^8^^,^^9^^,^^10^ These asymmetries are complex and multivariate traits influenced by a variety of factors, including age, gender, and intracranial volume (ICV).^9^^,^^11^ In addition, a variety of brain disorders, such as Alzheimer’s disease (AD), major depression (MD), schizophrenia (SZ), and autism spectrum disorder (ASD) are closely associated with altered asymmetry.^12^^,^^13^^,^^14^^,^^15^ Recently, researchers have paid more attention to the brains of newborns to uncover the origins of brain asymmetry.^16^^,^^17^ These studies greatly enhance our understanding of brain asymmetry, enabling easier exploration of the brain’s intrinsic properties. However, the methods currently in use for estimating asymmetry remain unsatisfactory. For example, most relevant studies use the asymmetry index (AI),^18^ obtained by comparing the LH and RH, as an indicator, but it only indicates the degree of lateralization without revealing the neuroanatomical details responsible for the lateralization.

**Figure 1:**
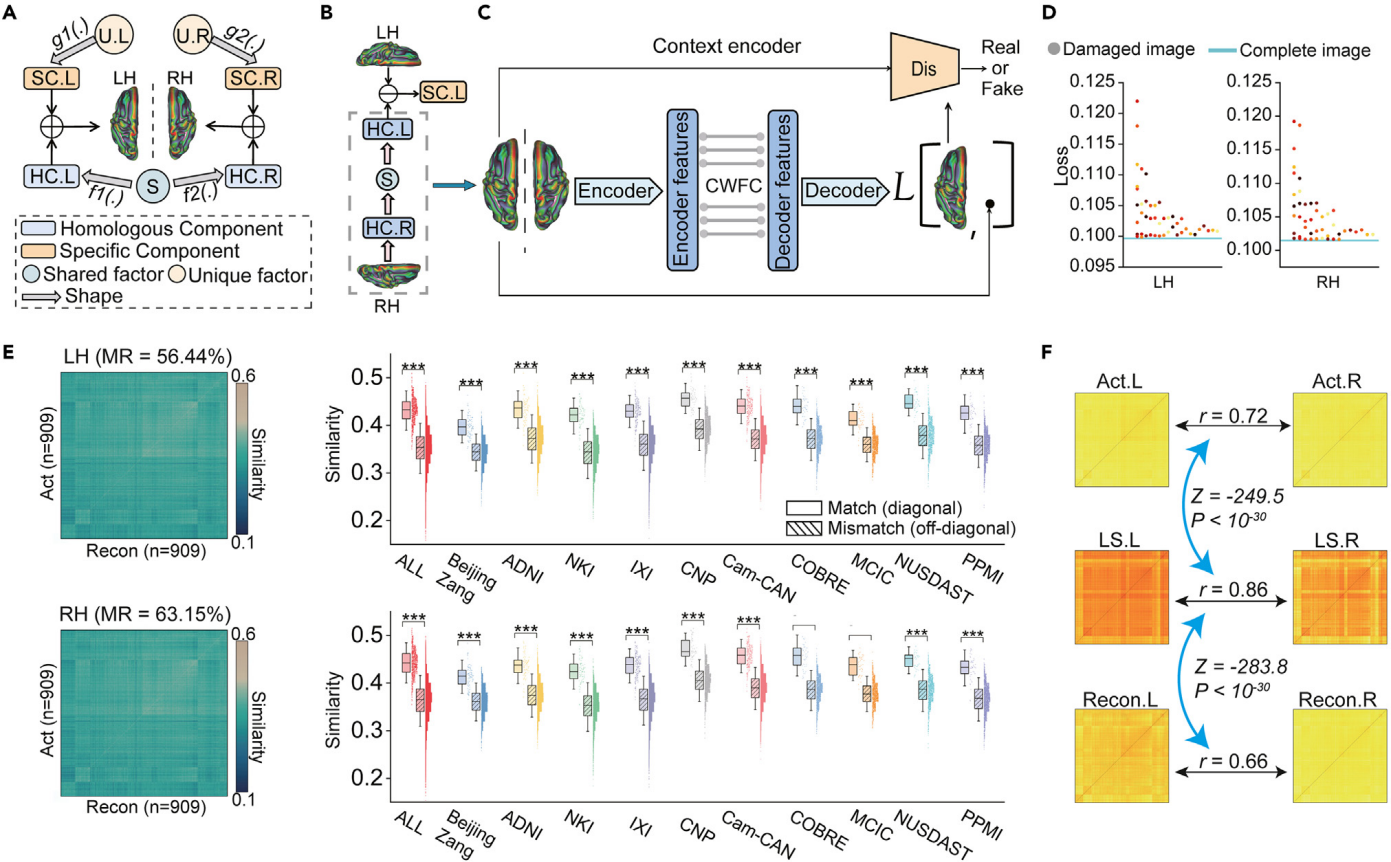
Training the DGNs to use one hemisphere to predict the opposite hemisphere (A) Brain hemispheres are composed of homologous and specific components. HC, homologous component; SC, specific component; S, shared factor; U, unique factor. *f1*(.) and *f2*(.) denote the mapping from S to HC. *g1*(.) and *g2*(.) denote the mapping from U to SC. (B) Using CE to establish mapping between HCs of the LH and RH, which follows the principle of hemisphere formation. (C) The CE architecture. We trained two CE models to implement the prediction of LH to RH and RH to LH. Dis, discriminator; CWFC, channel-wise fully connected. (D) Averaged reconstruction loss across subjects when the model is fed with “virtual lesion” images. Each point represents a class of damaged images (n = 58). The blue line indicates reconstruction loss observed with complete images fed into the model. (E) The similarity between all pairs of reconstructed and actual hemisphere images. In the heatmaps, diagonal values (n = 909) indicate similarity between matched images, and off-diagonal values (n = 825,372) indicate similarity between unmatched images. MR, match rate. The data in the box range from 25% to 75%. Error bars indicate standard deviation (SD) ×1.5. ∗∗∗p < 10^−30^ using a two-sample t test. (F) RSA between the LH and RH. Black arrows indicate the Spearman correlation analysis between RDMs. Blue arrows indicate comparisons between correlation coefficients using Steiger’s z test. Act indicates the actual hemisphere, Recon indicates the reconstructed hemisphere, and LS indicates the latent space (feature map in the bottleneck of the model). See also Figures S1 and S2.

According to brain development and lateralization,^1^^,^^6^^,^^7^ the process of lateralized specialization can be considered the coupling of shared factors and unique factors. Shared factors (such as most genes and stronger interhemispheric interactions) underlie the formation of both the LH and RH, while unique factors (such as asymmetric light, posture, and injury and inhibition of interhemispheric communication) dominate the direction of development of only one hemisphere. As a result, in real-world scenarios, the LH and RH are structured by homologous components and hemisphere-specific components (Figure 1A). We believe that uncovering hemisphere-specific components will provide deeper insights into brain asymmetries. However, with limited *a priori* knowledge, it is difficult to disentangle these two components directly.

Assuming an ideal situation in which the LH and RH are shaped only by shared factors, it is possible to establish an unambiguous mapping between them because they consist only of strongly dependent homologous components (similar to homotopy invariants in topology).^19^^,^^20^ When this ideal mapping function is applied to the real world, we can leverage the actual LH (or RH) to reconstruct a “fake” RH (or LH) consisting of homologous components. Afterward, by comparing the actual hemispheres with the “fake” hemispheres, we can infer hemisphere-specific components derived from the asymmetrical influence of unique factors. Here, we aimed to uncover the connections between homologous components and later reveal hemispheric specializations.

Deep generative networks (DGNs) provide reliable tools for image composition, which can accurately perform style transformations,^21^ such as transforming facial photos into cartoon styles. Currently, they have been widely applied in the synthesis of high-quality brain images.^22^^,^^23^ In addition, DGNs have the powerful ability to learn to recover images by viewing corrupted examples without ground truth data training. For instance, in the absence of clean samples, they can achieve effective denoising by building a mapping between pairs of noisy images^24^ or by self-supervised learning on a single noisy image.^25^ In the LH and RH, their homologous components are strongly dependent, while their specific components are relatively independent. Thus, even without ground-truth data (e.g., when the brain is shaped only by shared factors), it is possible to capture homologous components from empirical neuroimages by training DGNs.

In pursuit of both high-quality generated images and biologically reliable mapping, we modified a state-of-the-art DGN, the context encoder (CE), to implement complex nonlinear transformations. CEs are unsupervised learning algorithms that can generate the content of an arbitrary image region conditioned on its surrounding environment.^26^ They are jointly trained by minimizing both a reconstruction loss and an adversarial loss,^27^ which can produce much sharper results. CE-inspired methods are conducive to capturing biological representations from medical imaging.^28^^,^^29^ When CE is applied to brain magnetic resonance imaging (MRI), the generated images are more likely to have a biologically semantic structure rather than just a visual appearance.

In this study, we used a large sample of gray matter (GM) maps (n = 2,852), an indirect measure of the complex structure of neurons, to train and validate the models. The goal of the models was to convert one side of the hemisphere to fit the opposite hemisphere as well as possible. During training, to find the optimal solution, the models continuously approximated inherent dependent patterns between homologous components. The computational process essentially follows the principle of brain lateralization, which focuses on meaningful features, encodes them into a common latent space (similar to shared factors), and later decodes them in another space (Figure 1B).

We trained one CE to implement the conversion from LH to RH and another CE to implement conversion from RH to LH. We used the difference between the reconstructed and actual hemispheres as a biological indicator to estimate the neuroanatomical specialization in the hemisphere. Evidence has suggested that model failures can be informative.^30^ Difference between predicted and chronological age,^31^ for example, is of value for assessing normal aging and disease.^32^^,^^33^ This provides strong support for the reliability of our proposed biological indicators.

First, we verified that the trained models are biologically plausible and that our proposed hemispheric specialization features are stable and reliable. Second, we demonstrated that hemispheric specialization features are important biological indicators that are differentially related to age, sex, disease, environment, and cognition. Then, we demonstrated that the spatial distribution of hemispheric specialization features is plausible and found that the LH and RH have unequal contributions to hemispheric specialization. Furthermore, through a comprehensive analysis, we identified some crucial regions that account for most of the variation in hemispheric specialization and that control and drive the hemispheric specialization process.

## Results

### The reconstructed hemispheres are biologically plausible

We used 1,372 healthy subjects from 10 datasets (Table 1) to train two CE models (Figure 1C) and verified the biological plausibility of the model from several aspects.

**Table 1:** .Information about the datasets

Dataset	Type	Sex (M/F)	Age	Training set	Validation set
Beijing Zang	healthy	76/122	18–26	120	78
IXI	healthy	242/308	18.9–86.3	342	208
CNP	healthy	71/66	21–50	75	52
Cam-CAN	healthy	316/325	18–90	390	251
COBRE	healthy	66/23	18–65	45	44
	SZ	62/12	19–65	–	74
MCIC	healthy	64/28	18–60	55	37
	SZ	78/26	18–61	–	104
ADNI	healthy	86/101	56.3–89.1	105	82
	AD	87/67	55.7–90.4	–	154
NKI	healthy	115/76	4–85	120	71
NUSDAST	healthy	53/43	14–64	60	36
PPMI	healthy	68/37	31–81	60	45
	PD	61/37	38–83	–	98
ICBM	healthy	35/43	19–85	–	78
MSC	healthy	5/5	24–34	–	10
Yale-TRT	healthy	4/4	27–56	–	8
QTIM	healthy	23/27	21–29	–	50
Overall		1,512/1,350	4–90.4	1,372	1,480

M, male; F, female. See also Table S3.

First, we estimated whether the CE-based models were applicable to the brain MRI data. We iteratively fed the “virtual lesion” hemispheric images, in which a region is masked, into the trained model and then calculated the reconstruction loss. We found that the loss values of the virtual lesion images were all higher than the loss values of the complete images (Figure 1D), indicating that each brain region contributes positively to the generation of images. This result suggests that the model focused on the whole brain and did not neglect information in any region. In addition, we evaluated the quality of optimization during training using an independent dataset, International Consortium for Brain Mapping (ICBM). As the number of training epochs increases, the quality of the reconstructed images improves in both the training set and the independent validation set. Thus, the models are not overfitted and are robust across data domains.

Then, we examined whether the CE models captured the semantic information in the brain. We calculated the pairwise similarity between all pairs of actual and reconstructed images (heatmaps in Figure 1E). The results showed that within-individual similarity was significantly higher than between-individual similarity both in the combination of all datasets and within each dataset (p values < 10^−30^, two-sample t test; boxplots in Figure 1E). In addition, we conducted ablation experiments to analyze the rationality of the network architecture. The results showed that, when the discriminator was removed, the reconstructed image was unable to be precisely matched with the respective actual image, and the within-individual similarity was notably diminished (Figure S1). Thus, our CE models make it easier to capture the semantic information related to individual identity and retain it in the reconstructed brain.

Furthermore, we tested whether our model tended to capture the shared factors underlying the LH and RH. We constructed the representational dissimilarity matrices (RDMs) using the actual and reconstructed hemispheres as well as the latent space of the models (extracted from the bottleneck of the models). The representational similarity analysis (RSA) showed that the similarity between the actual and reconstructed LH and RH was significantly lower than the similarity between the latent spaces of the LH and RH (z = −249.5 and −283.8, p values < 10^−30^, Steiger’s z test; Figure 1F). In addition, the interindividual similarity increased as the layers became deeper (p = 0, one-way analysis of variance [ANOVA]; Figure S2). Thus, our models simulated the inverse process of brain evolution, which automatically encoded the LH and RH into a relatively shared space.

Collectively, these findings demonstrate the plausibility of our trained CE models, which characterize all brain voxels, capture the idiosyncratic pattern of an individual, and follow the principles of brain development and lateralization.

### Distribution of hemisphere-specific neuroanatomical components

**Figure 2:**
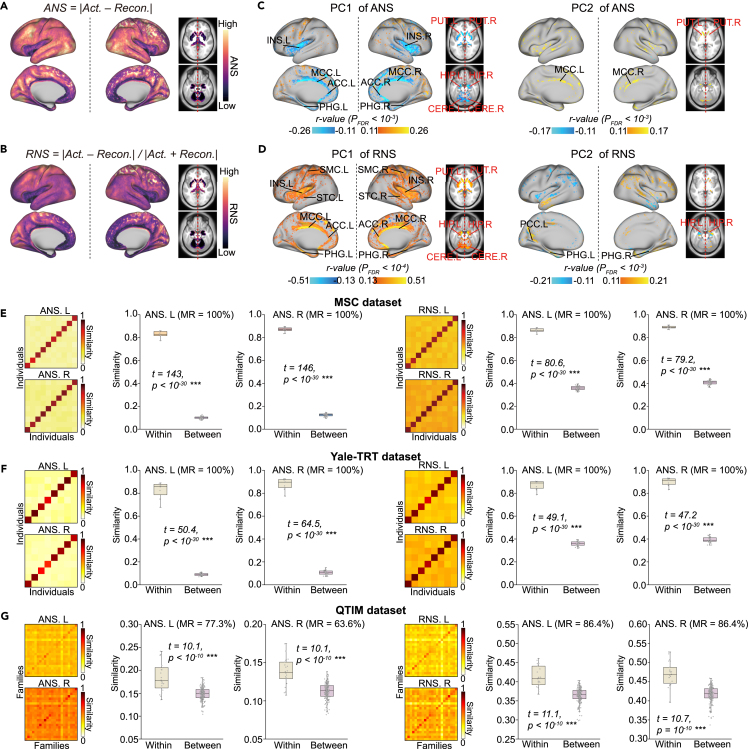
Distribution of ANS and RNS and validation of their reliability (A and B) Definition of ANS and RNS and visualization of ANS and RNS maps for the LH and RH (averaged across healthy people in the validation set, n = 909). (C and D) The main variant loci of ANS and RNS across individuals. Areas most relevant to the first two PCs of ANS and RNS (Pearson partial correlation analysis; thresholds are illustrated in the figure, and p values were corrected by FDR). In (A–D) the analyses were performed separately for the LH and RH, and we visualized them together. (E and F) Similarity between all paired ANSs or RNSs of the two scans in the MSC dataset and Yale-TRT dataset. Diagonal values indicate similarity within individuals (n = 10 and 8), and off-diagonal values (n = 45 and 28) indicate similarity between individuals. (G) The similarity between all pairs of ANSs or RNSs of the two families in the QTIM dataset. Diagonal values indicate similarity within families (n = 22), and off-diagonal values (n = 231) indicate similarity between families. The two-sided two-sample t test was used for between-group comparisons. The data in the boxes range from 25% to 75%. Error bars indicate SD × 1.5. INS, insula; ACC, anterior cingulate cortex; MCC, median cingulate cortex; PCC, posterior cingulate cortex; SMC, somatosensory and motor cortex; STC, superior temporal cortex; PHA, parahippocampal area; PUT, putamen; HIP, hippocampus; CERE, cerebellum. See also Figures S3‒S8 and Table S1.

Afterward, we used the trained models to calculate the hemispheric specialization of the remaining subjects. To better characterize hemispheric specialization, we applied two metrics: absolute neuroanatomical specificity (ANS) and relative neuroanatomical specialization (RNS), which indicate the amount and proportion, respectively, of specific components in a voxel of the LH or RH. We visualized the averaged ANS and RNS maps of the LH and RH across 909 healthy subjects from the validation set (Figures 2A and 2B; see Figure S3 for a flat surface). The ANS of the LH was higher than that of the RH, while there was no difference between the RNS of the LH and that of the RH (Figure S4). To facilitate the analysis of the biological properties of the ANS or RNS, we sorted the voxels in the LH or RH into 10 equal parts (regions) according to their ANS or RNS values. The mask for each part is shown in Figure S5.

Furthermore, we aimed to identify loci of variation in hemispheric ANS/RNS among individuals. To represent the major variation, we computed the first two principal components (PCs) of the hemispheric ANS/RNS maps across 909 healthy subjects from the validation set. We later calculated the correlation between PCs and ANS/RNS for each voxel (Pearson partial correlation using age, sex, and ICV as covariates; Figures 2C and 2D). The results suggest that the important loci were mainly in the somatosensory and motor cortices, insula, superior temporal cortex, cingulate cortex, subcortex, and cerebellum. Labeling of regions was performed according to the automated anatomy labeling (AAL) atlas^34^ and Glasser et al.^35^

### ANS and RNS are reliable bioindicators

Next, we verified the reliability of the ANS and RNS values. First, we tested whether our method is robust to various training samples. Since different individuals and groups have their innate and acquired characteristics, we re-partitioned the training and validation sets to train the models and computed ANS and RNS. Through this repeatability test, we found that the feature maps and variant loci we obtained from both experiments were essentially identical (Figure S6). This result somewhat suggests that our method is stable despite potential bias in the data.

Second, we tested whether ANS and RNS are robust over two scan sessions. We estimated the ANS and RNS of Midnight Scanning Club (MSC) and Yale Test-Retest (Yale-TRT) individuals and calculated pairwise similarity between all pairs of scan sessions (heatmaps in Figures 2E and 2F). We found that, for both ANS and RNS, the match rate (MR) was 100% (percentage of maximum values in each row that occur on the diagonal), and within-individual similarity was significantly higher than between-individual similarity (p values < 10^−30^, two-sample t test; boxplots in Figures 2E and 2F). This result suggests that the ANS and RNS are stable over multiple scans using the same scanner or different scanners.

Third, we tested whether ANS and RNS are heritable in relatives. We estimated the ANS and RNS of Queensland Twin IMaging (QTIM) individuals and calculated pairwise similarity between all pairs of families (heatmaps in Figure 2G). Within-family similarity was significantly higher than between-family similarity (p values < 10^−30^, two-sample t test; boxplots in Figure 2G). This result suggests that similar living environments lead to similar ANS and RNS, which is consistent with the conclusion that the degree of brain lateralization is heritable.^36^

Furthermore, we compared the robustness and heritability of our method with two commonly used methods. One of them is the AI,^18^ commonly applied to assess brain asymmetry, the other is a perturbation-based method^37^ that generates a saliency map (SM), commonly used for interpretive analysis of deep learning. For MSC and Yale-Retest, ANS showed a greater difference between within- and between-individual (or family) similarity than AI and SM, and for QTIM, both ANS and RNS show a greater difference than AI and SM (Figure S7). Moreover, in all three datasets, the MR of ANS and RNS is either greater than or equal to that of AI and SM (Table S1). This result suggests that our method is more stable and reliable in identifying interindividual variation, especially in identifying families. In addition, we visualized our proposed ANS/RNS map and SM of the three subjects (Figure S8) for easy and intuitive viewing.

Together, these results suggest that ANS and RNS are robust and biologically meaningful and may be used to represent brain “fingerprinting” and explain variations between individuals.

### ANS and RNS are associated with age and sex

Aging and gender all have a significant impact on brain neuroanatomy and asymmetry.^9^^,^^38^^,^^39^ In this section, to identify the biological characteristics of the hemispheric specialization features, we analyzed the relationship between the ANS and RNS with these phenotypes.

**Figure 3:**
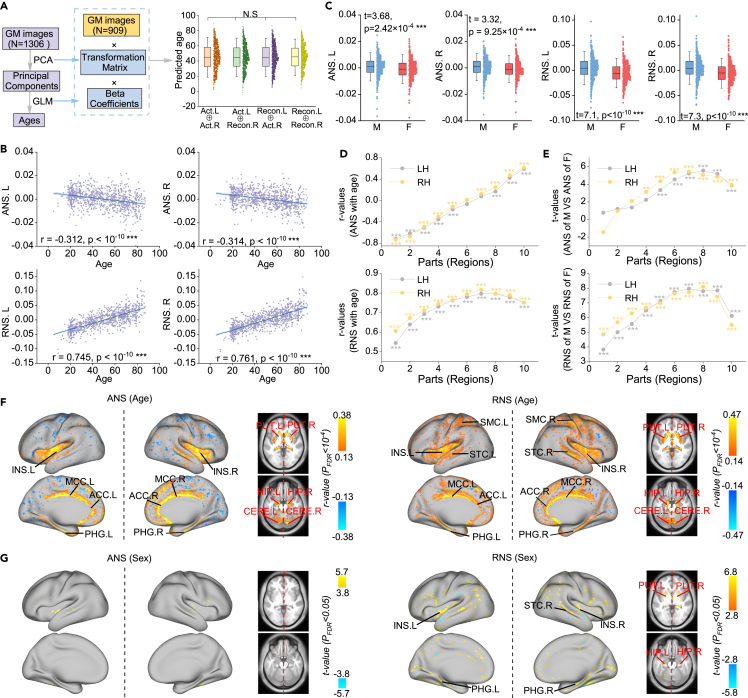
ANS and RNS are associated with age and sex (A) Predicted age (n = 903) based on actual or reconstructed hemisphere images. The transformation matrix of the principal-component analysis (PCA) and the beta coefficients of the regression model were estimated from the training set. Gray arrows indicate the flow of data, and blue arrows indicate the transfer of model weights. The symbol (×) denotes matrix multiplication. (B) Correlation between mean hemispheric ANS/RNS with age (n = 903, Spearman correlation analysis since the subjects’ ages did not obey a normal distribution). The shaded area represents the 95% confidence interval. (C) Differences between the mean hemispheric ANS/RNS of men (n = 486) and women (n = 417). Two-sided two-sample t test. (D and E) Correlation analysis with age and comparison with sex were performed separately in each of the 10 parts (Figure S5). ∗∗p < 0.01, ∗∗∗p < 0.001 with FDR correction. (F) The loci whose ANS/RNS had the most significant association with age. (G) The loci whose ANS/RNS differed most significantly between men and women. The data in the boxes range from 25% to 75%. Error bars indicate SD × 1.5. See Figure 2 for the full names of the regions. See also Figure S9 and Table S2.

Brain aging is generally associated with a loss of GM tissue, so GM can be used to accurately predict chronological age.^40^ We first examined whether the reconstructed brain preserved age-related semantic information. Participants in the training set were used to build dimensionality reduction and regression models, which were later applied to the actual and reconstructed images in the validation set (Figure 3A). We found no significant difference (p > 0.05, paired-sample t test) between the ages predicted from the actual and reconstructed images. Likewise, to demonstrate that the reconstructed images also preserve gender-related information, we used the training set to build a gender classification model, which was then applied to the actual and reconstructed images in the validation set. The classification performance of actual and reconstructed images is highly similar (Table S2). Thus, models have similar perceptions^41^ of actual and reconstructed images, indicating that the reconstructed images preserve the inherent patterns of the brain and have the potential to provide meaningful insights into the brain.

We calculated the overall (averaged across all voxels) and regional (averaged across the voxels within each part in Figure S5) ANS and RNS of the LH and RH for each of the healthy participants (6 individuals had no age and sex information; we only analyzed 903 individuals here). Then, we regressed gender and ICV from the ANS/RNS when analyzing the effect of age and regressed age and ICV from the ANS/RNS when analyzing the effect of gender. Interestingly, the overall ANS of the LH and RH showed a positive correlation with age (p values < 10^−10^, Spearman correlation; Figure 3B), whereas the overall RNS of the LH and RH showed a negative correlation with age (p < 10^−10^, Spearman correlation; Figure 3B). The overall hemispheric ANS and RNS were higher in males than in females (p values < 0.001, two-sample t test; Figure 3C). Figure 3D shows the correlations (r values) between age and each regional ANS/RNS, and Figure 3E shows the differences (t values) between males and females for each regional ANS/RNS. These line graphs all surprisingly indicated an almost upward trend rather than a random distribution, suggesting that the ANS and RNS may be used to guide the parcellation of the brain.

Moreover, we performed voxelwise analyses to identify loci closely associated with age and sex (Figures 3F and 3G). We found that the RNS was more sensitive to sex differences than the ANS, and both the ANS and RNS were sensitive to changes in age. Significant areas were located mainly in the somatosensory and motor cortices, insula, superior temporal cortex, cingulate cortex, subcortex, and cerebellum.

For comparison, we also calculated the association of AI with age and gender. We discovered that both ANS and RNS were more sensitive to changes in age compared with AI. RNS displayed greater sensitivity to changes in sex than AI, whereas ANS showed sensitivity to changes in sex comparable with AI (Figure S9). Thus, ANS and RNS can better explain inter-individual variation in hemispheric specialization caused by age and sex variation.

Together, these results demonstrate that the hemispheric ANS and RNS are meaningful biomarkers that are closely related to the basic human phenotype.

### ANS and RNS are associated with cognition, the environment, and disease

Individual cognitive function is affected by brain lateralization,^11^^,^^39^ the external environment is an important cause of brain lateralization,^42^ and alterations in the typical brain lateralization lead to disease.^11^^,^^13^^,^^15^^,^^42^ In this section, to further examine whether the ANS and RNS are accurate representations of hemispheric specialization, we analyzed the association between the ANS and RNS with cognitive measures, demographic information, and brain disease.

**Figure 4:**
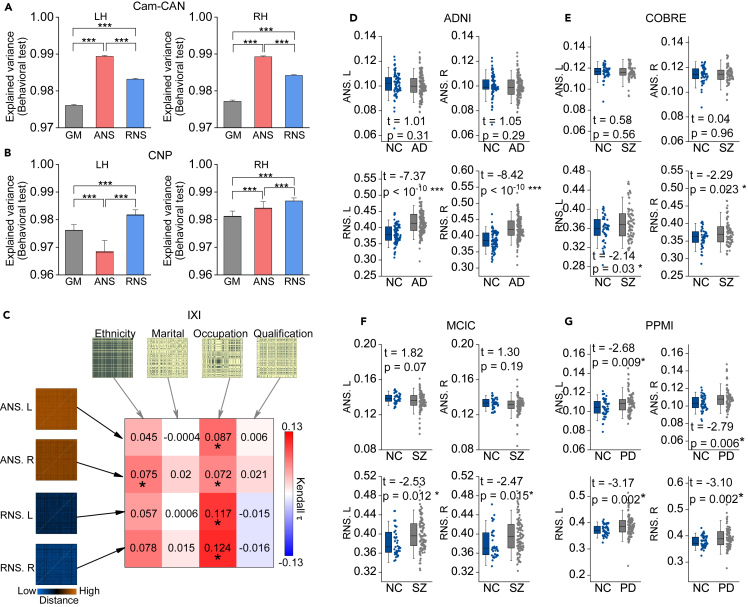
ANS and RNS are associated with cognitive ability, environmental factors, and disease (A and B) Behavioral performance and intelligence measures explained by hemispheric ANS, RNS, and actual GM. The data used in (A) (n = 232) and (B) (n = 50) were collected from the Cam-CAN and CNP datasets, respectively. The explained variance is calculated by a multivariate explanatory model. Paired-sample t tests were used for comparisons between groups. The error bar indicates the SD × 1.5. ∗∗∗p < 10^−10^. (C) RSA between ANS/RNS and demographic information in the IXI dataset (n = 206). Kendall rank correlation was used to estimate the similarity between RMDs. ∗p < 0.05 with 5,000 permutation tests. (D–G) Comparison of overall hemispherical ANS/RNS between patients and healthy individuals from the ADNI (154 AD, 82 NC), COBRE (74 SZ, 44 NC), MCIC (104 SZ, 37 NC), and PPMI (98 PD, 45 NC) datasets (two-sided two-sample t test). The data in the boxes range from 25% to 75%. Error bars indicate SD × 1.5. ∗p < 0.05. NC, normal control; AD, Alzheimer’s disease; SZ, schizophrenia; PD, Parkinson’s disease. See also Figure S10.

The Cambridge Centre for Ageing and Neuroscience (Cam-CAN) database provides behavioral measures of participants, and the Consortium for Neuropsychiatric Phenomics (CNP) database provides intelligence tests of participants. We used a multivariate explanatory model^43^ to estimate the association between the ANS and RNS with these metrics and used the actual hemisphere as a baseline. The results indicated that both ANS and RNS of the LH and RH can accurately explain the cognitive abilities of individuals (p < 0.001, 5,000 permutation tests). Moreover, except for the ANS of the LH in CNP, the remaining ANS and RNS can explain cognitive abilities better than actual GM (p values < 10^−10^, paired sample t test; Figures 4A and 4B).

Next, we explored the relationship between ANS/RNS and demographics (e.g., ethnicity, marriage, occupation, and qualification) of participants in the Information eXtraction from Images (IXI) dataset using RSA.^44^ We found that occupation was associated with both ANS and RNS of the LH and RH, and ethnicity was associated with ANS of the RH (p < 0.05, 5,000 permutation tests). This result suggests that ANS and RNS are indeed under the influence of some specific environmental factors.

To investigate whether diseases affected the ANS and RNS, we analyzed patient data from four datasets, including AD, SZ, and Parkinson’s disease (PD). We also calculated the overall hemispheric ANS and RNS for patients and then compared healthy individuals and patients in each database separately. The results showed that only the overall hemispheric ANS of PD patients was significantly higher than that of healthy subjects (p < 0.05, two-sample t test on the Parkinson Progression Marker Initiative [PPMI]; Figure 4G), but the overall hemispheric RNS was significantly higher in all patients (p < 0.05, two-sample t test; Figures 4D–4G). In the comparison of regional ANS/RNS, we found more between-group differences (Figure S10). The regional ANS/RNS alterations were not identical for different types of diseases.

These findings further suggest that the ANS and RNS can capture interindividual variation caused by cognitive function and environmental factors and abnormalities in brain structure. In the future, they may be used as biomarkers to infer the characteristics of individuals.

### ANS and RNS obey spatial autocorrelation

**Figure 5:**
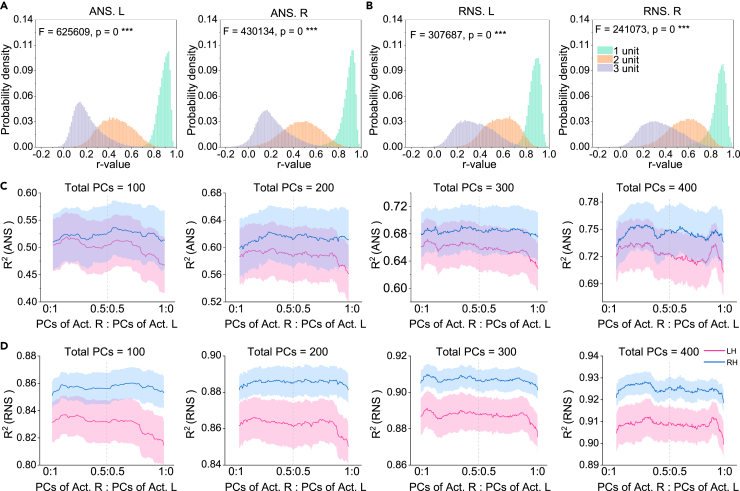
Spatial autocorrelation properties of ANS and RNS and unequal effect of the LH and RH on ANS and RNS (A and B) Correlation of the ANS/RNS of each voxel (n = 70,000) with the ANS/RNS of the voxel at a distance K from it. The unit of distance is the length of a voxel (1.5 mm). In this study, K = 1, 2, and 3. The x axis represents the R value, and the y axis represents the probability density. One-way ANOVA was used for comparisons between groups. (C and D) Regression analysis between the hemispheric actual GM and the hemispheric ANS/RNS. The independent variable is the PC of the actual GM, and the dependent variable is the mean ANS/RNS within each part (Figure S5). The x axis represents the ratio between PCs of the LH and PCs of the RH in the independent variable, where the total number of PCs is constant. The y axis represents the mean R^2^ across 10 parts, which evaluates how well the model fits. The shaded part indicates the standard error (SE). See also Figure S11.

Brain features possess spatial autocorrelation (SA).^45^ Due to SA, values of brain feature in spatially proximal regions tend to be more similar than values of spatially distant regions. To demonstrate whether the spatial distributions of the ANS and RNS maps are biologically plausible, we explored the SA of the ANS and RNS. We randomly selected approximately 70,000 voxels and calculated the partial correlation (age, gender, and ICV as covariates) between each voxel and its surrounding voxels at distances ranging from 1–3. The results showed that, for both ANS and RNS, the correlations decreased significantly with increasing distance (p values = 0, one-way ANOVA; Figures 5A and 5B), suggesting that the spatial distributions of ANS and RNS maps follow SA. The correlation between the ANS/RNS of voxels and the GM density of voxels around them also decreased with increasing distance (p values < 10^−10^, one-way ANOVA; Figure S11).

### LH and RH structural patterns have unequal contributions to hemispheric specialization

We have demonstrated the reliability and plausibility of the hemispheric ANS and RNS in several ways. Here, we analyzed the association between brain structural patterns and hemispheric specialization. Multiple linear regression was used to map the representations of actual GM to the hemispheric ANS/RNS. As shown in Figure 2A, we first extracted the PCs of the actual LH and RH (the transformation matrix was estimated using the training set, which was later applied to the validation set). Then, keeping the total number of PCs as N (in this study, N = 100, 200, 300, or 400), we concatenated the PCs of the LH with those of the RH in different proportions (ranging from 0:1 to 1:0 with a step of 1/N) and then used each combination to fit the ANS/RNS for each of the 10 parts (Figure S5). Figures 5C and 5D show the averaged R^2^ of fitting of each combination. The models fitted both the ANS and RNS of the RH better than those of the LH (the blue line is higher than the red line). Additionally, the model’s fit to the ANS/RNS of the LH decreased more significantly when the ratio between PCs of the RH and PCs of the LH was close to 0:1 (the smallest R^2^ values of the LH were all located on the rightmost side of the x axis). These findings suggest that the formation of RH specialization is more likely to be influenced by innate brain patterns and that biological factors conducive to the RH specialization are distributed throughout the brain, while biological factors leading to LH specialization are primarily distributed in the LH.

### Important areas for controlling hemispheric specialization

**Figure 6:**
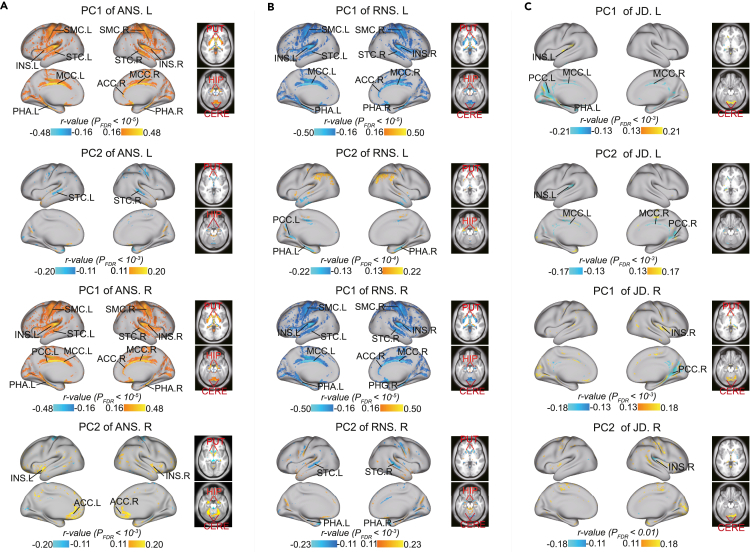
Visualization of important regions that control and drive hemispheric specialization (A and B) Correlation between the first two PCs of the hemispherical ANS/RNS maps with each actual voxel. (C) Correlation between the first two PCs of the hemispherical JD maps with each actual voxel. Pearson partial correlation analysis was used (age, sex, and ICV were used as covariates). The p values were corrected by FDR. In different subgraphs, we used different thresholds to locate the most significant voxels. See Figure 2 for the full names of the regions. See also Figures S12 and S13.

We performed voxelwise analysis to identify loci that were closely related to hemispheric specialization. As we did for Figures 2C and 2D, we computed the first two PCs of ANS/RNS maps of the LH and RH across healthy participants (n = 909). Next, we calculated the correlations between two PCs with the actual GM density of each voxel (Pearson partial correlation using age, sex, and ICV as covariates). Figures 6A and 6B show the loci most significantly correlated with the ANS and RNS, respectively. The results suggest that the somatosensory and motor cortices, insula, superior temporal cortex, cingulate cortex, parahippocampal gyrus, cerebellum, and subcortical regions are the most important areas for controlling hemispheric specialization. These significant regions largely overlapped with the major variant loci in the ANS and RNS (Figures 2C and 2D and 3E and 3F).

Furthermore, we identified the loci closely associated with the process of hemispheric specialization. First, we calculated the deformation field using a nonlinear registration that transformed the reconstructed hemispheres into the corresponding actual hemispheres. We then computed the Jacobi determinant (JD) of the deformation field as an index of local expansion or contraction (Figure S12).^46^ The resulting JD map can be used to estimate the dynamics leading to hemispheric specialization formation (how to drive homologous components to form an actual hemisphere). Consistent with the above analysis, we computed the first two PCs of the LH and RH JD maps and calculated the correlation between PCs with the GM density of each voxel. The significant areas (Figure 6C) were largely consistent with those in Figures 6A and 6B, suggesting that they play a dominant role in driving hemispheric specialization. In addition, we found that PCs of the JD map had a smaller effect on age and gender (Figure S13).

## Discussion

Our study proposed a new method using a self-supervised DGN to reveal hemispheric specializations in the brain. The results suggest that our method is stable, reliable, and plausible and is able to capture individual variation characterizing a wide range of individual traits. We expect that our method will provide inspiration for the characterization of brain function and structure. Next, we discuss our findings from the joint perspective of methodology, neuroscience, and applications.

Deep learning provides powerful and promising tools for neuroimaging research because it can learn high-level and abstract representations of data using complex nonlinear transformations.^47^ Deep learning methods have been widely used for medical image classification, segmentation, alignment, and other tasks.^48^ Moreover, recently, researchers have focused more on using deep learning methods to automatically capture and identify high-level biomarkers. Two related studies demonstrate that the latent variables and residuals generated by variational autoencoders (VAEs) accurately captured the interindividual variation in the functional MRI (fMRI) data.^49^^,^^50^ Chavas et al.^51^ suggested that β-VAE can help reveal unknown cortical folding patterns. Zhao et al.^52^ proposed a new autoencoder model to obtain representations of brain function. Aglinskas et al.^53^ leveraged contrastive VAEs (CVAEs) to better characterize the neuroanatomical variation specific to ASD. These studies and our work share the same inspiration: using self-supervised deep learning methods to obtain biologically meaningful markers. However, instead of encoding the entire brain image with an autoencoder-based model, our work uses masked image modeling (MIM) to create mapping between the two hemispheres to reveal biologically specific components of each part. It has been suggested that the learned representation in autoencoders is likely to be the compression of image content, while MIMs have a deeper semantic understanding of the scene.^26^^,^^54^ Biomedical data collection is time consuming and laborious, so acquiring training targets for complex tasks is often impossible, but the results demonstrate that the MIMs are able to learn the information we expect without target samples. This study supports and extends the previous theory to some extent, showing that MIM approaches are promising for deep characterization of the brain. Another difference is that most autoencoder-based representation learning methods use the latent space as a new biomarker, which is informative but hard to interpret. However, in this work, the representations restored in the original image space are still informative and easy to understand, which facilitates subsequent analysis at the region or voxel level.

In addition, we note that our workflow is theoretically similar to that of the activity flow mapping method.^55^ Activity flow mapping shows that task-evoked activity in one region can be predicted by the functional connectivity (FC)-weighted sum of task-evoked activity in other regions and that the similarity between predicted and actual activity can be used to estimate interregional information transfer and reveal disease-related features.^56^^,^^57^ Similarly, our study essentially uses one region to explain another region, after which the unexplained variance is considered to be a biological indicator. Neurons in the brain are all interdependent,^58^ which provides a biological possibility to uncover the mapping between brain regions. Our work and previous studies demonstrate that, when the established mapping function is biologically plausible, its derivatives are likely to reveal high-level biological features. In terms of performance, however, deep learning methods have greater potential to fit intricate interactions between neurons than simple linear methods.

To estimate voxelwise or vertexwise lateralization, most studies have used significant warping and spatial smoothing to force the brain into a symmetric space, thereby establishing a left-right correspondence. Then, the lateralization is quantified by comparing the original image and the flipped image. However, even if the brain is spatially symmetric, this does not guarantee a biological correspondence because the global pattern of the brain is continuously reshaped under the influence of external factors.^6^ In addition, a fair comparison usually requires the condition that the LH and RH are within the same data space. However, the structure and function of the brain already exhibit significant asymmetries at birth.^16^^,^^59^ This implies that the neurobiological substrates of the LH and RH (similar to the developmental functions *f1* and *f2* in Figure 1A) are already heterogeneous at the beginning of life, some of which are unexplainable, confounding many attempts at alignment.

In contrast, in our work, the generator reveals the complex dependencies between homologous components of the two hemispheres, and the discriminator facilitates learning the distribution of target spaces conditioned on source spaces. Our approach implements semantic transfer, which expresses the content of one hemisphere in the style of the opposite hemisphere. This process eliminates gaps between different spaces and establishes a biological correspondence between the LH and RH, thus allowing a fair comparison.

In this study, we proposed two metrics for estimating hemispheric specialization: ANS and RNS. Our results demonstrate that they both encode much biological information, such as age, gender, disease, environmental factors, and cognitive function. However, through a comprehensive comparison, we believe that the RNS is more reliable and informative. The RNS is calculated from the ratio of the ANS to GM density, and this operation mitigates the interference caused by some confounding factors. For example, the ANS was negatively correlated with age, while the RNS was positively correlated with age. This is because the RNS eliminates the effect of GM density decreasing with age. Furthermore, this finding suggests that the proportion of specific components underlies brain asymmetry and dominates the formation of interindividual differences.

Evidence has shown that the cingulate gyrus, insula, subcortex, and cerebellum have strong anatomical and functional connections to brain structures and possess important hub-like properties.^60^^,^^61^^,^^62^ In the current study, these areas had relatively low ANS and RNS, implying their robust structure and biological symmetry, but their ANS and RNS accounted for most of the variation between individuals and correlated strongly with sex and age. Furthermore, these regions controlled the overall pattern of hemispheric specialization. Our results support and extend previous findings suggesting that these areas play an important role in the establishment of hemispheric specialization.

In this work, our main goal is to suggest that DGNs are promising tools for the study of brain asymmetry. We used only a simple network backbone consisting of several convolution neural network (CNN) blocks for validation. In the future, researchers can modify the network backbone and the loss function according to the needs of the study, further improving the accuracy of the results. Examples include using a hybrid framework of CNN and self-attention^63^ to focus on both local and global information or adding a regularization module to capture more semantic information. In addition, the results provided a preliminary indication that our proposed ANS and RNS capture and encode a variety of biologically meaningful information. They can be extended and applied to a wide variety of research as complementary tools of neuroscience, such as for revealing the properties of certain regions of interest (ROIs), exploring the development and aging of the brain, and diagnosing brain diseases.

Although our method is oriented toward brain neuroanatomy, it can be easily transferred to fMRI analysis by simply altering the architecture of the network. For instance, for building generators and discriminators using graph neural networks to analyze the FC networks of the brain. The analytical process in our work is not limited to the study of hemispheric specialization but can be used to study the specialization of any region as long as there is a sound neuroscientific basis for it. For example, by establishing a mapping between cortical and subcortical tissue, we can infer their specialization components and processes.

This study has some limitations that can be improved in the future. Firstly, machine learning techniques are susceptible to bias, leading to discrimination against specific groups or populations.^64^^,^^65^ For example, studies have shown that predictive models perform better for white European/Americans and worse for ethnic minorities.^66^ Therefore, in future work, we need to further demonstrate whether the method is unbiased for different samples. Secondly, machine learning methods are vulnerable to adversarial attacks, meaning that even a small perturbation to the data can cause a significant alteration in the output.^67^^,^^68^ In the future, we need to improve the stability of the model against adversarial attacks. Moreover, we mainly performed a global analysis that only considered the absolute value of the ANS or RNS. However, the positive and negative nature of the model error also has important biological implications, which have been demonstrated in brain age-related studies.^69^ We will later clarify this issue to further improve the interpretability of our method.

In conclusion, we gathered multiple neuroimage databases and trained a self-supervised DGN that leverages one hemisphere to predict the opposite hemisphere. Based on the model prediction error, we propose ANS and RNS to describe hemispheric specialization. The results demonstrate that this approach is feasible and promising in multiple aspects. Our study not only explores brain asymmetry from a new perspective but also sheds new light on the application of DGN to neuroimaging.

## Experimental procedures

### Resource availability

#### Lead contact

Requests for further information and resources should be directed to the lead contact, Tianyi Yan (yantianyi@bit.edu.cn).

#### Materials availability

This study did not generate new unique reagents.

#### Data and code availability

The Alzheimer’s Disease Neuroimaging Initiative (ADNI) and PPMI datasets are publicly available from https://ida.loni.usc.edu/login.jsp. The Center of Biomedical Research Excellence (COBRE), Mental Illness and Neuroscience Discovery Institute Clinical Imaging Consortium (MCIC), and Northwestern University Schizophrenia Data and Software Tool (NUSDAST) datasets are publicly available from http://www.schizconnect.org/. The Beijing Zang, ICBM, Nathan Kline Institute (NKI), and Yale-TRT datasets are publicly available from http://fcon_1000.projects.nitrc.org/. The IXI dataset is publicly available from http://brain-development.org/ixi-dataset/. The Cam-CAN dataset is publicly available from http://www.cam-can.com/. The CNP dataset is publicly available from https://openneuro.org/datasets/ds000030/. The MSC dataset is publicly available at https://openneuro.org/datasets/ds000224/. The QTIM dataset is publicly available from https://openneuro.org/datasets/ds004169/.

All analysis code is available on Zenodo^70^ (https://doi.org/10.5281/zenodo.10466961).

### MRI data

All neuroimaging data used in the study were T1-weighted MRI scans. We collected data on healthy people and patients from 14 publicly available datasets, made available via various data-sharing initiatives; there were a total of 2,852 samples with an age range of 4–90.4 years (Table 1). Among the datasets, 10 datasets were used to train the models and analyze the main results. One dataset was used to evaluate the generality of the model. Three datasets were used to evaluate within-scanner reliability, between-scanner reliability, and heritability. The imaging parameters are shown in Table S3.

The datasets used to train the model and analyze the main results include Beijing Zang, IXI, Cam-CAN,^71^ COBRE, MCIC,^72^ ADNI,^73^ NKI, NUSDAST,^74^ CNP,^75^ and PPMI.^76^ According to the age and gender distribution and sample size of each dataset, 1,372 healthy subjects were randomly selected to train the models. The remaining 909 healthy subjects and 430 patients were used for the analysis of the main results.

The ICBM with 78 healthy subjects was used to evaluate the convergence of the model and the quality of the reconstructed images during training. When the loss value and the quality of the reconstructed image stabilized, we stopped the training and saved the model.

The MSC^77^ and Yale-TRT^78^ datasets were used to evaluate the stability of the same individual in different scans. Each health participant in MSC underwent 2 scanning sessions on separate days, which were collected using a same 3-T Siemens Trio. Each healthy participant in Yale-TRT underwent scans over 4 sessions approximately 1 week apart, using the 2 identically configured Siemens 3-T Tim Trio scanners: 2 sessions used “scanner A,” and the other 2 sessions used “scanner B.” We used two scans of 10 subjects in MSC and one scan from “scanner A” and one scan from “scanner B” of 8 subjects in Yale-TRT.

The QTIM^79^ was used to evaluate the heritability among family members. It is a multimodal neuroimaging dataset of young adult twins and siblings. In our study, we randomly selected 47 subjects from 22 families.

### Data preprocessing

We performed minimal preprocessing on the data. The T1-weighted images were segmented into separate tissue classes. All GM density maps were normalized to the standard Montreal Neurological Institute (MNI) template with a 1.5 × 1.5 × 1.5 mm^3^ voxel resolution (values were normalized to 0 to 1). These steps were implemented in FMRIB Software Library (FSL). We then used a GM probability mask (GM probability threshold = 0.15) to extract voxels corresponding to GM tissue, removing the noise and artifacts at the edge of the GM tissue. Finally, we split the GM into the LH and RH along the x = 0 axis in the transverse section and cropped each hemisphere to remove the black background.

Data were excluded if an anatomical scan failed to complete the tissue segmentation procedure or if tissue segmentation was inaccurate. The number of subjects stated above was determined after quality assessment (Table 1).

### Deep learning model

The generative adversarial network (GAN) training strategy is to define a game between two competing networks. The generator network (G) transforms data from one space to another. The discriminator network (D) receives either a generated sample or a real data sample and must distinguish between the two. G is trained to produce more realistic images, eventually confusing D. The CE is a self-supervised MIM based on the GAN architecture.^26^ Its G consists of an encoder, which captures the context of an image into a compact latent feature representation, and a decoder, which uses this representation to generate the missing image content. D allows G to capture more details and semantic information, improving the quality of the image reconstruction.

In this study, the encoder consists of four repeated down-blocks of a 3D CNN layer, a 3D batch normalization (BN) layer, and a rectified linear unit (ReLU). The decoder consists of four repeated up-blocks of a 3D deconvolution layer, a 3D BN layer, and a ReLU. A channel-wise fully connected layer is used to connect the encoder and the decoder, allowing each unit in the decoder to analyze the entire image content. D consists of four CNN layers and one classification layer.

The LH and RH are described by the following formulations:

(Equation 1) $$x_{LH}=h_{LH}+s_{LH},x_{RH}=h_{RH}+s_{RH}$$

where *h* indicates homologous components, and *s* indicates specific components. Both $h_{LH}$ and $h_{RH}$ originate from shared factors; thus, they are not independent:

(Equation 2) $$p\left(h_{LH}|h_{RH}\right)\neq p\left(h_{LH}\right),p\left(h_{RH}|h_{LH}\right)\neq p\left(h_{RH}\right)$$

However, $s_{LH}$ and $s_{RH}$ are derived from factors unique to the LH and RH, respectively, so they are independent:

(Equation 3) $$p\left(s_{LH}|s_{RH}\right)=p\left(s_{LH}\right),p\left(s_{RH}|s_{LH}\right)=p\left(s_{RH}\right)$$

As a result, the network can still estimate the *h* in one hemisphere by understanding the opposite hemisphere.

The objective of the network is to use the LH or RH to predict the RH or LH, respectively, thus naturally revealing the innate mapping between $h_{LH}$ and $h_{RH}$. The loss function is a combination of reconstruction loss and adversarial loss. The reconstruction loss is responsible for capturing the overall structure of the missing region and coherence regarding its context but tends to average together the multiple modes in predictions. We used a voxelwise L2 distance as the reconstruction loss. Take using the LH to predict the RH as an example:

(Equation 4) $$L_{recon}={\left\|x_{RH}-G\left(x_{LH}\right)\right\|}_{2}$$

(Equation 5) $$X=\left[x_{LH}⊕x_{RH}\right]$$

where X is the whole brain. ⊕ indicates the concatenation operation.

The adversarial loss, on the other hand, tries to make the prediction look real and has the effect of picking a particular mode from the distribution.^27^ Inspired by the Wasserstein GAN (WGAN), we add a gradient penalty to our adversarial loss, which can improve the stability of training, eliminate problems such as mode collapse, and provide meaningful learning curves useful for debugging and hyperparameter searches.^80^ The adversarial loss for CEs is

(Equation 6) $$L_{G}=-D\left(x_{RH}\right)$$

(Equation 7) $$L_{D}=D\left(x_{\text{RH}}\right)-D\left(G\left(x_{\text{LH}}\right)\right)+\lambda {\left[{\left\|\nabla_{x}D\left(\hat{x}\right)\right\|}_{2}-1\right]}^{2}$$

(Equation 8) $$x=\varepsilon x_{\text{RH}}+\left[1-\varepsilon \right]G\left(x_{\text{LH}}\right)$$

where ∇ stands for gradient calculation. ε is a random number between 0 and 1. We define the overall loss function as:

(Equation 9) $$L=\lambda_{rec}L_{rec}+\lambda_{adv}\left[L_{G}+L_{D}\right]$$

In this work, $\lambda_{rec}$ is 0.9 and $\lambda_{adv}$ is 0.1. We trained two CE models by the same strategy, one using the LH to predict the RH and the other using the RH to predict the LH. The models were trained on four NVIDIA Tesla P100 graphics processing units (GPUs). The training epoch was 3000 when the reconstruction reached stability. Learning rate is 10^−4^, batch size is 16.

### The measure of hemispheric specificity

We fed the LH (or RH) image into the trained model and obtained the reconstructed RH (or LH) image. The hemispherical ANS map is represented by a voxelwise distance between the actual and the reconstructed image.

(Equation 10) $$ANS_{i}=abs\left(Act_{i}-Recon_{i}\right)$$

The index *i* indicates LH or RH. abs(·) denotes absolute value. In the training process, the objective is to reduce the distance between the outputs and the objects. Therefore, we do not consider the positive or negative of the difference between the reconstructed and the actual voxel.

Furthermore, we calculated the RNS map, estimating the proportion of specific components in the hemisphere.

(Equation 11) $$RNS_{i}=abs\left(\left(Act_{i}-Recon_{i}\right)/\left(Act_{i}+Recon_{i}\right)\right)$$

Afterward, to minimize non-biological variation introduced by different scanners and acquisition protocols, we used the Combining Batches of Gene Expression Microarray Data (ComBat) method^81^^,^^82^ to harmonize ANS and RNS values across scanners. The analyses in this study were all based on the harmonized ANS and RNS.

To explore whether the biological information described by the different ranges of ANS/RNS is varied, we divided the voxels into different parts. According to the average ANS or RNS map across individuals, we ranked voxels in descending order and divided them into 10 equal parts. For example, the first part corresponds to the voxels with the top 10% ANS or RNS, and the rest can be understood in the same manner.

### Comparison methods

To demonstrate the superiority of our approach, we compared two commonly used methods. The first is the AI, which has been widely used in previous studies of brain structural asymmetry. AI is defined as the ratio (LH−RH)/(LH+RH), ranging from −1 to 1, and a positive AI means a leftward asymmetry.

The second is a perturbation-based approach,^37^ widely used for interpretive analysis in deep learning. It masks or disturbs each pixel or region of the image in turn and observes the predicted changes, creating an SM. Here, a 3D sliding window with a size of 5 and a step of 2 was applied to occlude the image. A counter was used to track voxel occlusions. An SM is generated by dividing the cumulative predicted change by the counter.

### Verification of robustness, generalization, and heritability

To verify that the reconstructed images capture the intrinsic characteristics of each individual and not just a rough description of the brain shape of the group, we calculated the similarity between each reconstructed image and the corresponding actual image as well as the noncorresponding actual images. The image was first reshaped into a one-dimensional vector. Pearson correlation analysis was used to calculate the similarity between vectors.

We used the “virtual lesion” method to verify that our model accounts for the characteristics of all brain regions rather than only using some regions to establish mapping. The AAL atlas^34^ was used to define the ROIs. We masked one ROI (assigned a value of 0 to the voxels within the region) and then input the damaged image into the trained model to obtain the reconstructed image. Each ROI performed the above step.

To verify that the ANS and RNS are reliable indicators, we analyzed the stability of the same individual in different scans collected by the same or different scanners. To verify that ANS and RNS can be inherited, we analyzed the consistency among family members, including twins and siblings. The Pearson correlation coefficient was used to estimate the similarity within and between subjects and families.

### Principal-component analysis

Principal-component analysis (PCA) was used to reduce the dimensionality of features and to indicate major interindividual variation. For the actual hemispheres (GM maps), the transformation matrices of the PCA were estimated by the individuals in the training set and then were applied to individuals in the validation set. For the ANS, RNS, and JD maps, the transformation matrix obtained by 99% of the subjects in the validation set was used to calculate PCs for the remaining 1% of subjects. PCA was performed separately on the LH and RH.

To identify loci of individual variability related to anatomical specificity, we calculated the first two PCs that account for the most variation in the ANS, RNS, or JD maps and then correlated the PC loadings with the ANS, RNS, or JD values for each voxel.

### Regression analysis

Regression models were used to map actual hemispheres to age or ANS and RNS. When predicting ages, PCA was used to reduce the dimensions of the GM maps for both the LH and RH. We then concatenated the PCs of the LH and RH, explaining 50% of the variation, and used a linear regression model to establish the mapping between PCs and age. These steps were performed on the individuals in the training set. After training, the transformation matrix of the PCA and the weights in the linear regression model were used to predict the age of healthy individuals in the validation set. To explore whether the reconstructed images can properly depict brain aging, we applied four sets of images to predict ages: (1) $Act_{LH}⊕Act_{RH}$, (2) $Act_{LH}⊕Recon_{RH}$, (3) $Recon_{LH}⊕Act_{RH}$, and (4) $Recon_{LH}⊕Recon_{RH}$. *Act* denotes the real image. *Recon* denotes the reconstructed image. The subscript denotes the index of the hemisphere. ⊕ indicates the concatenation operation.

When predicting ANS and RNS, we also applied the transformation matrix from the training set to reduce the dimension of the actual GM within the validation set. Then, we mapped the PCs of actual GMs to ANS or RNS using multiple linear regression models and measured fittings according to R^2^. Specifically, age, sex, and ICV information were regressed out from PCs and ANS or RNS. We concatenated the PCs of the actual GM of the LH and RH in different proportions, ranging from 0:1 to 1:0 while keeping the total number N of PCs unchanged. For example, a ratio of 0.5:0.5 indicates concatenating the top N/2 PCs of the LH and top N/2 PCs of the RH; a ratio of 0:1 indicates choosing the top N PCs of the RH and no PCs of the LH. Afterward, each combination of PCs was fitted to the mean ANS/RNS of each of the 10 parts (Figure S5).

### RSA

We used RSA^44^ to examine the associations between neuroanatomical features and the associations between neuroanatomical features and demographic information. We collected the demographic variables from the IXI database, which described the ethnicity, occupation, marriage, and qualification of the subjects. The RDMs of neuroanatomical features were calculated by correlation distance (1 minus the correlation coefficient). For demographic variables, the subject distance was 0 if categorical variables matched (e.g., same ethnicity) and 1 otherwise. Considering that the demographic variables are discrete, we used Kendall rank correlations to estimate the similarity between the RDMs of the demographic variables and the RDMs of the neuroanatomical features. Spearman rank correlation was used to estimate the similarity between the RDMs of neuroanatomical features.

### Variance component model

We used a multivariate variance component model^43^ to link anatomical features and behavioral measures. The behavioral measures were collected from the Cam-CAN and CNP datasets. In Cam-CAN, participants completed a battery of behavioral tasks to assess their cognitive functions in the aspects of executive function, emotional processing, motor function, and memory.^71^ Our study focused on five tasks: fluid intelligence test, face recognition, picture priming, proverb comprehension, and emotion expression recognition. In CNP datasets, participants took part in the Wechsler Adult Intelligence Scale III (WISN-III) test, which included letter-number sequencing (LNS), matrix reasoning (MR), and vocabulary test (VOC).^75^

We first regressed age and gender from these phenotypic measures, which were then quantile normalized. The multivariate variance component model is shown as follow:

(Equation 12) Y=E+U

where *Y*, *E*, or *U* is the matrix with a size of *S* × *H*. *S* is the number of individuals, and *H* is the number of behavioral measures (*H* = 5 for Cam-CAN and *H* = 3 for CNP). *Y* contains the *H* processed behavioral measures for all *S* individuals. *E* and *U* represent common environmental factors and unique environmental factors, respectively. $vec\left(E\right)∼N\left(0,\sum_{e}⊗F\right)$ and $vec\left(U\right)∼N\left(0,\sum_{e}⊗I\right)$. *F* is a similarity matrix, where *F*(i, j) encodes the anatomical feature (GM, ANS, or RNS) similarity of subjects *i* and *j*. vec(.) is the matrix vectorization operator. ⊗ is the Kronecker product of matrices. *I* is an identity matrix, $\Sigma_{e}\in R^{H\times H}$ is a common environmental covariance matrix, and $\Sigma_{u}\in R^{H\times H}$ is the unique environmental covariance matrix, which is to be estimated from *F* and *Y*. The variance explained by the anatomical features, denoted by *M*, is computed as

(Equation 13) $$M=\left(Tr\left(\sum_{e}\right)\right)/\left(Tr\left(\sum_{e}\right)+Tr\left(\sum_{u}\right)\right)$$

where Tr(.) is the trace operator. *M* measures how much inter-individual behavioral variability is explained by inter-individual anatomical feature variability.

To facilitate statistical difference between the variance explained by two different metrics, we repeated the above steps *S* times, removing one sample in each calculation. The result *M*_*-i*_ of each time represents the variance explained from whole datasets without subject *i*. Then, a set of *M* values was used for comparison between groups.

### SA

To explore whether the spatial distribution of the ANS/RNS was spatially autocorrelated, we analyzed the similarity between voxels at different distances. We first sampled approximately 70,000 voxels at random. We then used Pearson partial correlation to calculate the similarity of a voxel with neighboring voxels at distances from 1 to 3 units (a basic unit is 1.5 mm in this study). We chose the front, back, top, bottom, left, and right voxels of a voxel as its neighboring voxels and averaged them before correlation analysis. One-way ANOVA was used to compare neighboring voxels at different distances.

### Deformation-based morphometry

We performed deformation-based morphometry analysis to estimate the internal dynamics of hemispheric specialization. Specifically, we applied a nonlinear warping (using inverse-consistent diffeomorphic image registration implemented in Advanced Normalization Tools [ANTs]) to transform each reconstructed LH or RH into its corresponding actual hemisphere. The resulting deformation field described how much each voxel was moved from the reconstructed hemisphere to match the corresponding original hemisphere. We then calculated the JD of the deformation field as an index of local expansion or contraction.^46^ This estimate captured the local expansion or atrophy required to morph the reconstructed hemisphere to the corresponding actual hemisphere.

### Statistical analysis

A two-tailed paired-sample t test was used to compare different indicators in a set of data. A two-tailed two-sample t test was used to compare different datasets. One-way ANOVA was used for comparison between multiple groups. Spearman’s rank correlation was used to calculate the association between age and anatomical features. Multiple comparisons were corrected by false discovery rate (FDR). Steiger’s z test was used to compare the correlation coefficients (r values).^83^

## Supplemental information

Document S1. Figures S1–S13 and Tables S1–S3

Document S2. Article plus supplemental information
