# Supplementary material for: Using a deep generation network reveals neuroanatomical specificity in hemispheres
Source: Patterns (N Y). 2024 Feb 12;5(4):100930. doi: 10.1016/j.patter.2024.100930 (PMC11026975; doi:10.1016/j.patter.2024.100930)
Supplement: Document S1. Figures S1–S13 and Tables S1–S3 [file mmc1.pdf]

Patterns, Volume *5*

Supplemental information

Using a deep generation network reveals neuroanatomical specificity in hemispheres

Gongshu Wang, Ning Jiang, Yunxiao Ma, Dingjie Suo, Tiantian Liu, Shintaro Funahashi, and Tianyi Yan

Supplemental Items

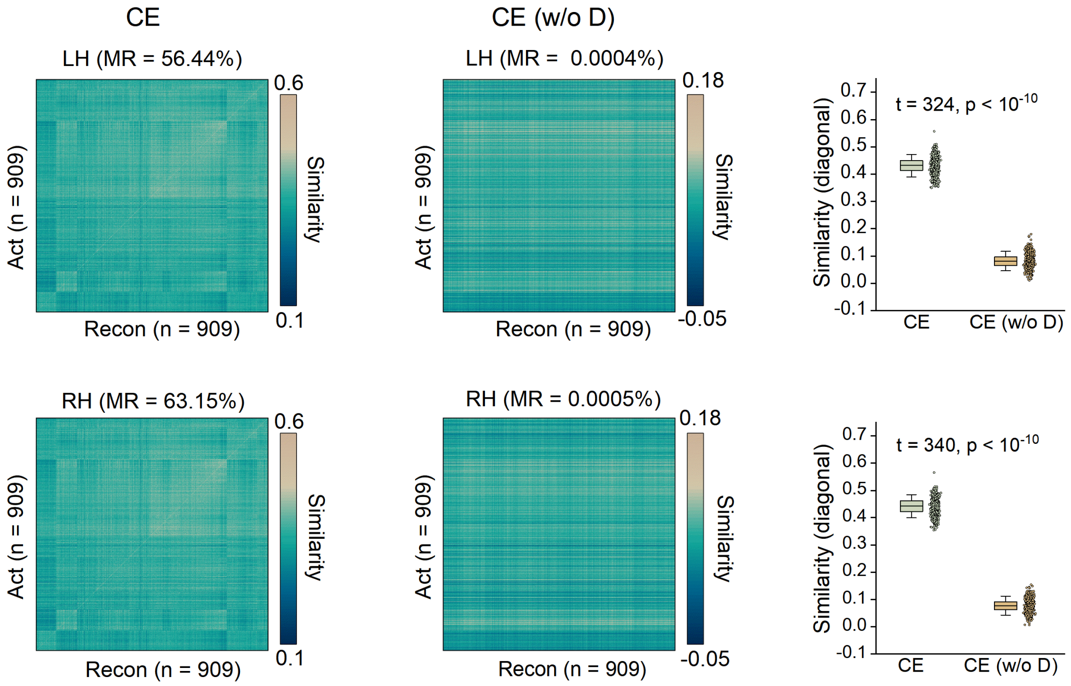

Figure. S1. Ablation experiment, related to Figure 1. When removing the discriminator in CE, the reconstructed image fails to match the corresponding real image, resulting in a significant reduction in similarity. The values in the heat map indicate the Pearson correlation coefficients between the images. Paired samples t-test for between-group comparisons. Error bars indicate SD×1.5. Act: actual; Recon: reconstructed; MR: matching rate; w/o: without; D: discriminators

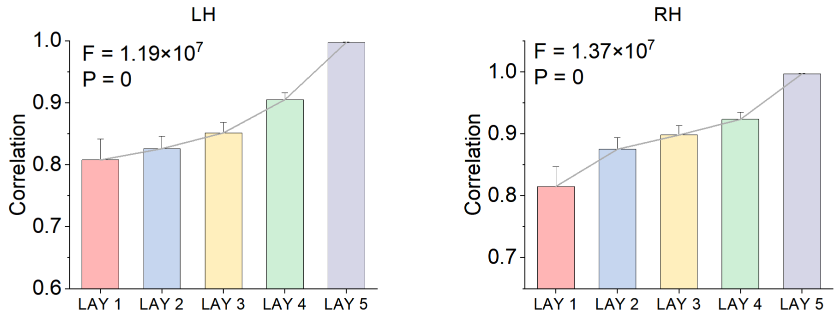

Figure S2. Inter-individual similarity calculated from the feature maps in the models, related to Figure 1. Data from healthy individuals in the validation set (n = 909). 'LAY' denotes the CNN layer in CE. LAY 1 to 4 denote the feature maps extracted from each of the four layers in the encoder, and LAY5 denotes the feature maps extracted from the bottleneck. Error bars indicate SD×1.5. ANOVA for comparisons between multiple groups.

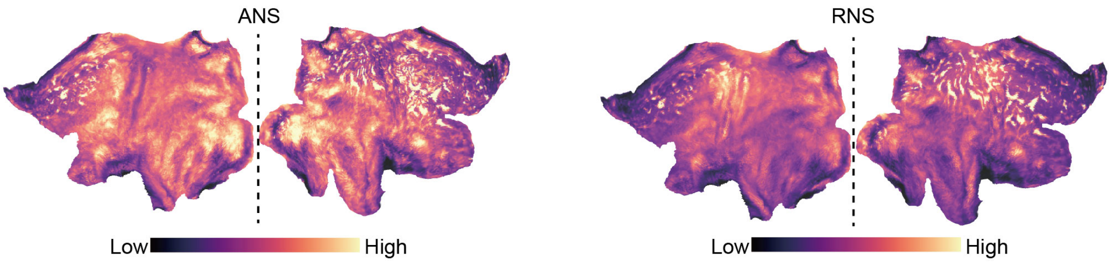

Figure S3. Average hemispheric ANS and RNS visualization in surface space, related to Figure 2.

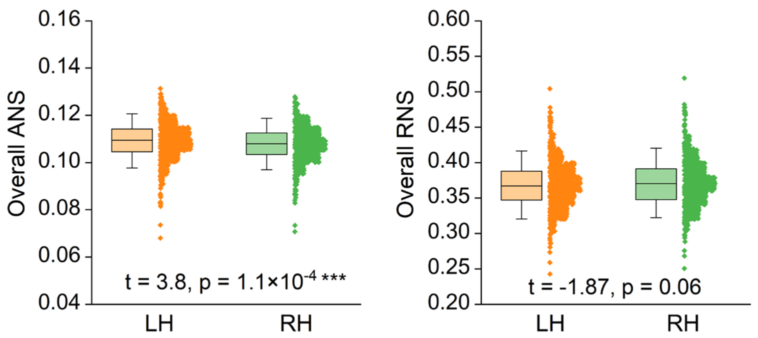

Figure. S4. Comparison between the overall ANS/RNS of the left and right brain, related to Figure 2. Paired samples t-test for comparison between groups. Error bars indicate SD×1.5. *** indicates $p < 0.001$.

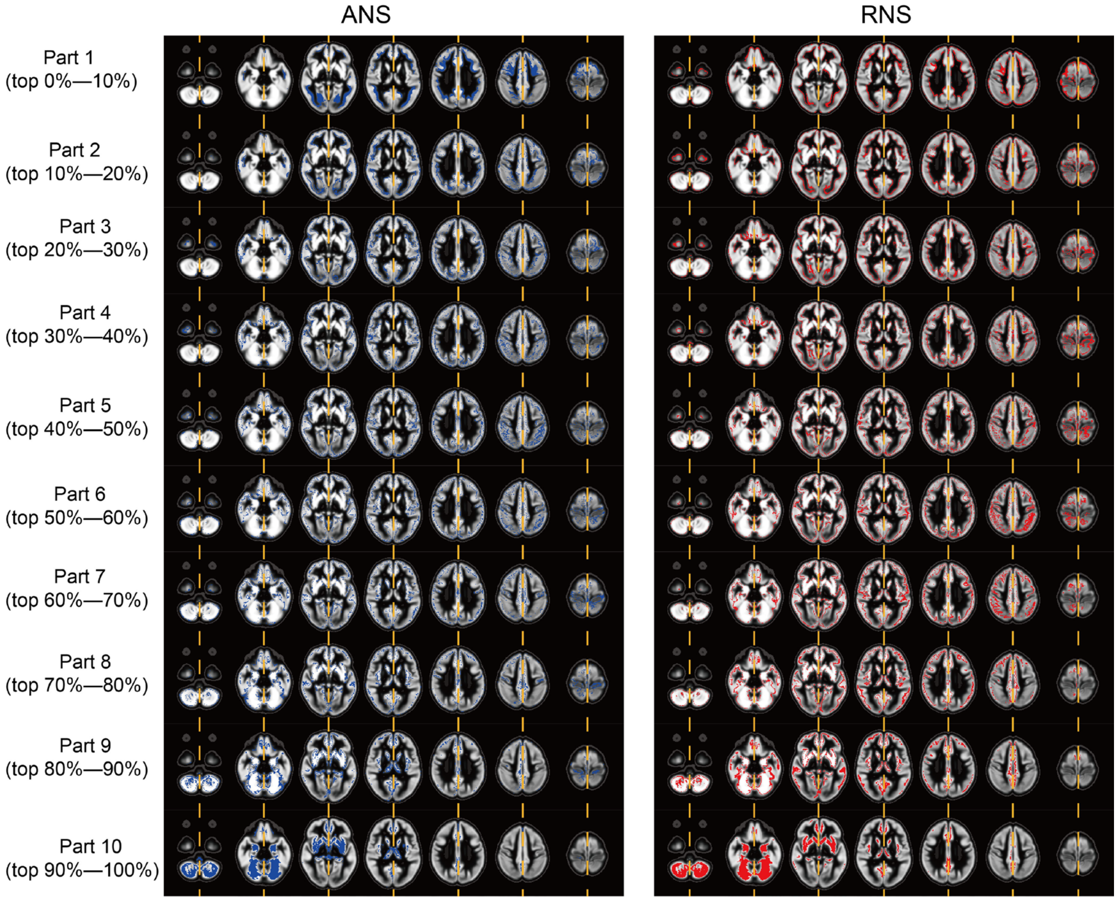

Figure S5. Voxel grouping based on ANS or RNS, related to Figure 3 and 5. According to the value of ANS or RNS, we sort the voxels and divide them into 10 equal parts(regions). For example, region 1 includes voxels in the top 10% of the ANS/RNS ranking, and region 10 includes voxels in the bottom 10% of the ANS/RNS ranking. Of note, the grouping of voxels is performed separately in the LH and RH, but we put the LH and RH together to visualize.

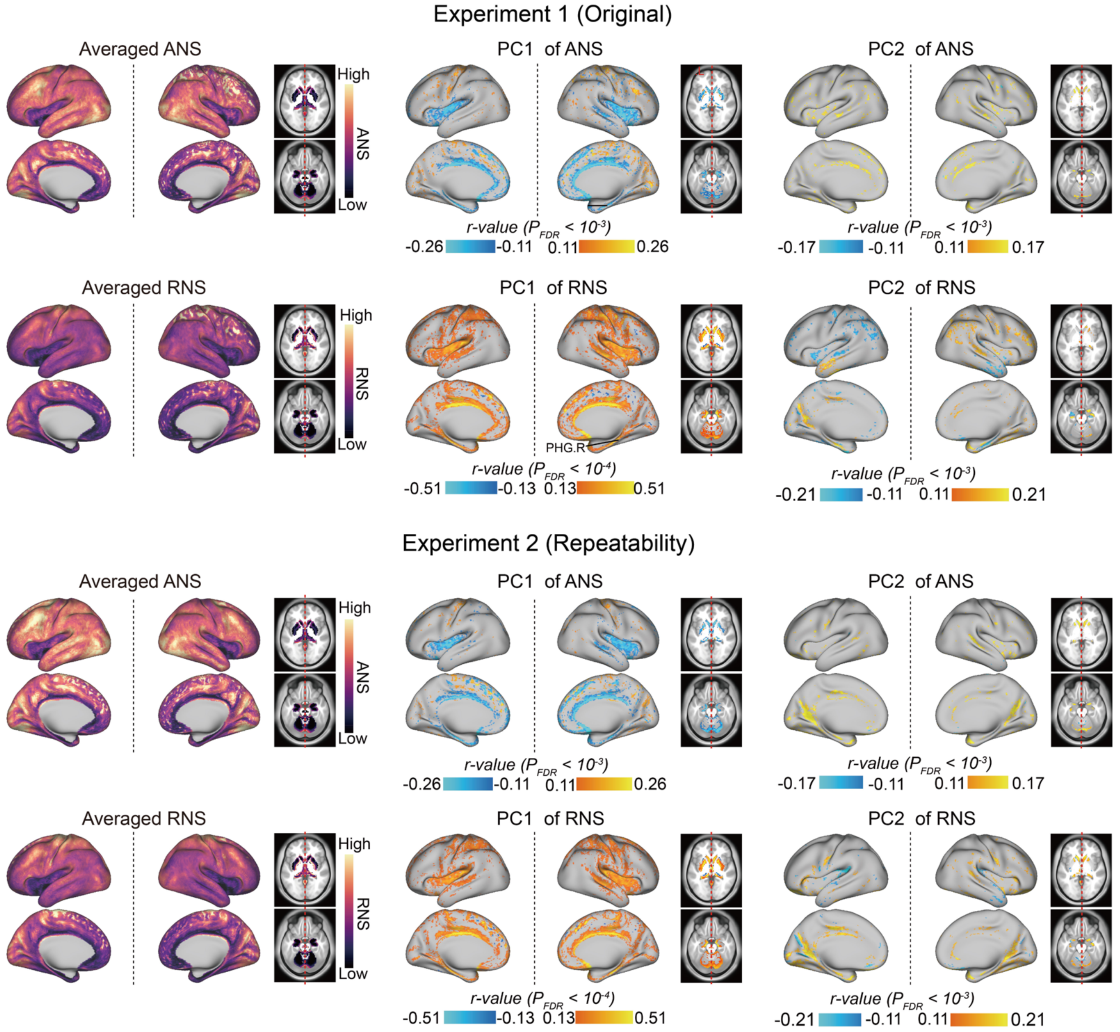

Figure S6. Repeatability testing, related to Figure 2. We reallocated the training (n = 1194) and test sets (n = 1095) from all samples and attempted to prevent overlap between the new and original sets (examples from the initial test set were included in the new training set). After that, we retrained the CE model and recalculated the ANS and RNS. The results demonstrate the robustness of our approach, as the new ANS and RNS maps and variant loci are largely consistent with those of the originals.

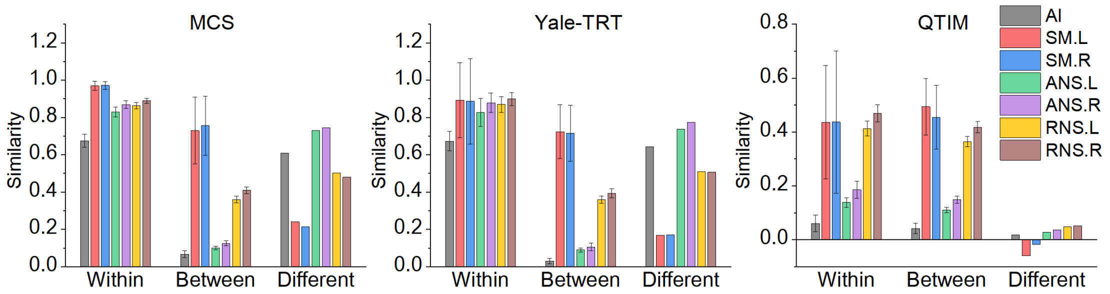

Figure S7. Comparison of ANS and RNS with asymmetric index and Saliency Map, related to Figure 2. Similar to Fig. 2E, F and G, we calculated the stability of the AI and SM across multiple scans and the heritability of asymmetric index among family members. Two-sample t-test for between-group comparisons. 'Different' denotes average 'Within' minus average 'Between'. AI: asymmetric index. SM: saliency map obtained by interpretable method. Error bars indicate SD×1.5.

Table S1. Accuracy of ANS, RNS, AI and SM for individual identification and family identification, related to Figure 2. AI: asymmetric index. SM: saliency map.

Dataset	AI	SM		ANS		RNS	
		LH	RH	LH	RH	LH	RH
MSC	100%	100%	100%	100%	100%	100%	100%
RETEST	100%	87.50%	87.50%	100%	100%	100%	100%
QTIM	27.27%	13.64%	13.64%	77.27%	63.64%	86.36%	86.36%

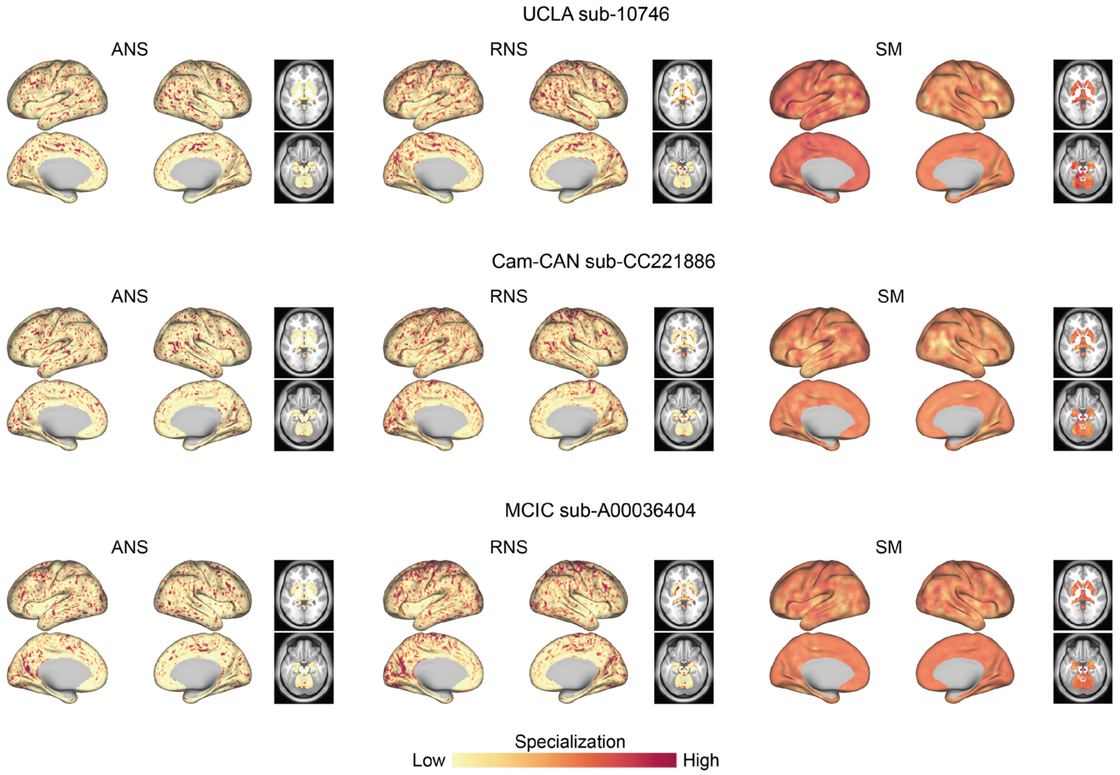

Figure S8. Visualization of our proposed ANS and RNS, as well as saliency map, related to Figure 2. The value in SM is 1 minus the normalized MSE, ranging from 0 to 1. Because, a smaller MSE indicates that the region has less influence on the contralateral hemisphere, suggesting a greater degree of asymmetry. SM: saliency maps

Table. S2. Performance of reconstructed and actual images for gender classification, related to Figure 3.

Feature	Accuracy	Precision	Recall	AUC
Act. ⊕ Act. R	55.92%	55.85%	53.52%	0.53
Act. L ⊕ Recon. R	54.04%	53.81%	50.46%	0.5
Recon. L ⊕ Aat. R	53.70%	51.43%	50.15%	0.5
Recon. L ⊕ Recon. R	54.26%	55.42	50.70%	0.5

⊕ indicates concatenation operation.

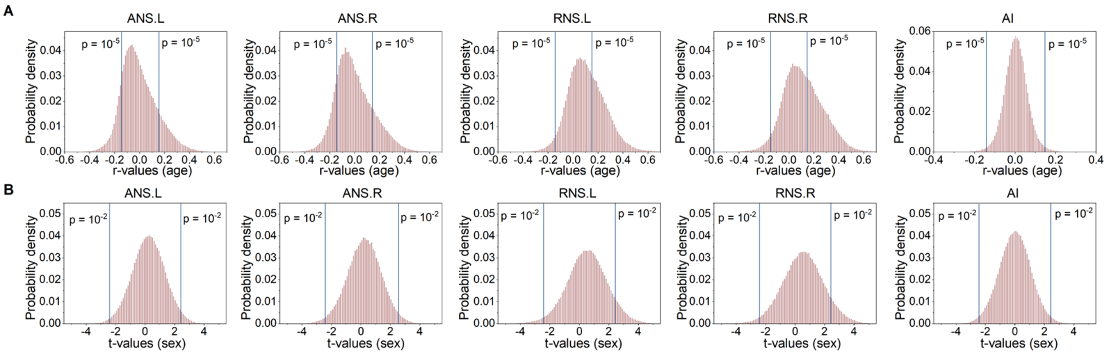

Figure S9. Comparison between ANS/RNS and AI in explaining inter-individual variability, related to Figure 3. We analyzed the **(A)** relationship between age and voxel-wise features (Spearman correlation analysis) and **(B)** compared voxel-wise features between males and females (two-sample t-test). The histograms represent the distribution of r-values and t-values. The blue lines in the figure indicate the thresholds, used to measure the significance of the results. AI: asymmetric index.

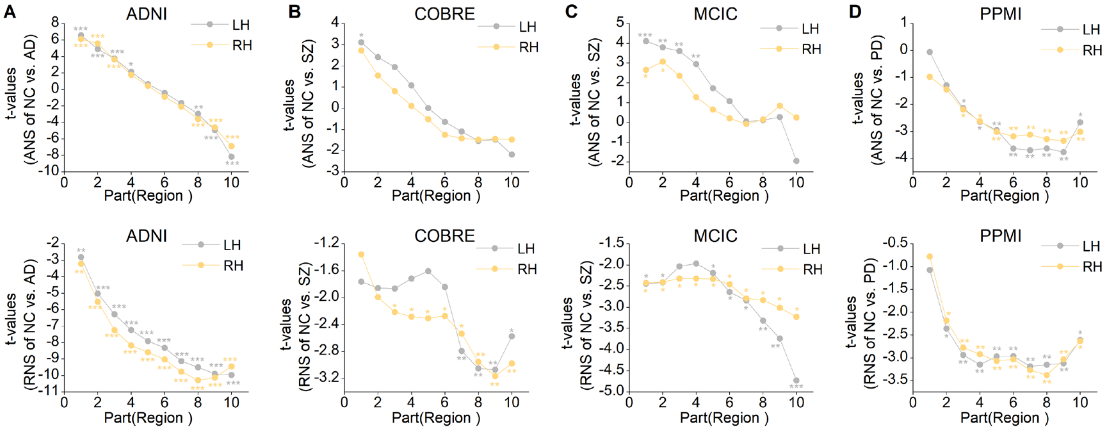

Figure S10. Comparison between the regional ANS/RNS of healthy people and patients, related to Figure 4. Two-sample t-test for between-group comparisons. The x-axis indicates the index of the region (Figure. S5). The y-axis indicates the t-value of the comparison between groups. ***: $p < 0.001$, **: $p < 0.01$, *: $p < 0.05$.

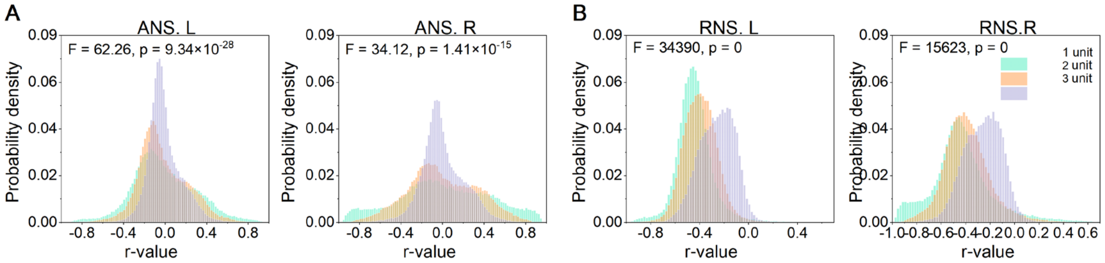

Fig. S11. Correlation of the ANS/RNS of each voxel with the GM density of the voxels at a distance K from it, related to Figure 5. The unit of distance is the length of a voxel (1.5mm). In this study, K = 1, 2 and 3. The X-axis represents the r-value, and the Y-axis represents the probability density. One-way ANOVA was used for comparison between groups.

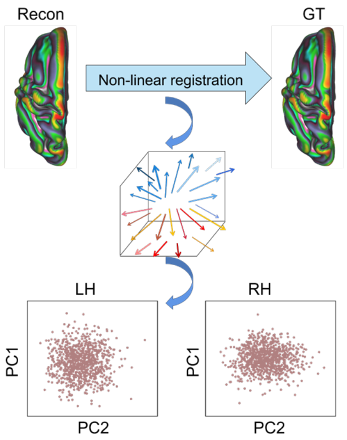

Figure. S12. Workflow of deformation-based morphometry analysis, related to Figure 6.

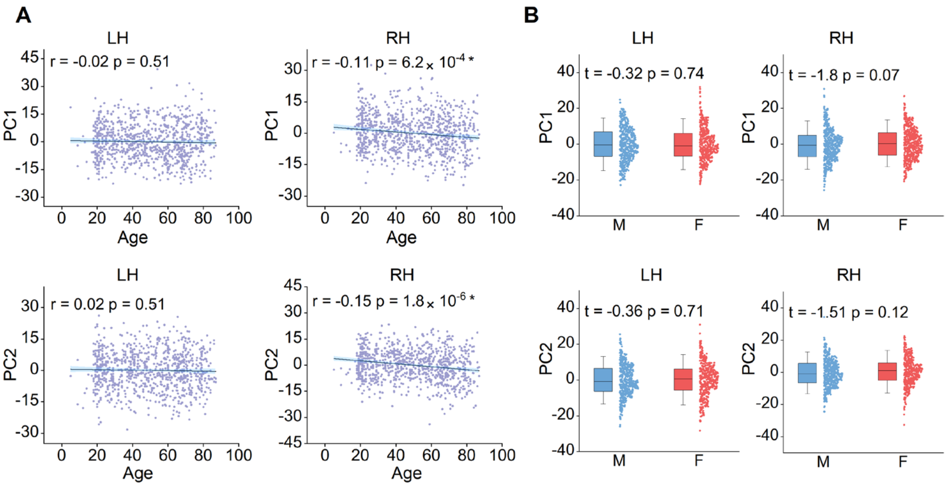

Figure S13. Association between Jacobi map with age and gender, related to Figure 6. **(A)** Pearson correlation analysis was used to calculate the similarity between age and PCs of the Jacobi map. The shaded area represents the 95% confidence interval. **(B)** Two-sample t-test for between-group comparisons. Error bars indicate SD×1.5.

Table S3. Imaging parameters, related to Table 1

Dataset	Strength	Repetition Time (ms)	Echo Time (ms)	Flip Angle	FOV (mm)	Voxel size (m^3)
IXI	1.5T or 3T	9600	4.6	8°		
COBRE	3T	2530	1.64	7°	256x256	1×1×1
Yale-TRT	3T	2400	1.18	8°		1×1×1
Cam-CAN	3T	2250	2.99	9°	256 × 240 × 192	1×1×1
CNP	3T	1900	2.26		250 × 250 × 250	1×1×1
MCIC	1.5T or 3T	2530	4.76	7°	256×256×128	1×1×1
NUSDAST	1.5T	9700	4			0.625×0.625×1.5
ADNI	3T	2300	2.98	9°	256×240	1×1×1.5
QTIM	4T	1500	3.35		230	0.9×0.9×0.9
MCS	3T	2400	3.74	8°		0.8×0.8×0.8

The imaging parameters of PPMI are available at http://www.ppmi-info.org. The imaging parameters of Beijing zang, NKI and ICBM are not available.
